# Out with the old - advancements and shortcomings of the updated 9th edition of the Tumor, Node, Metastasis (TNM) classification system for lung cancer

**DOI:** 10.36416/1806-3756/e20250022

**Published:** 2025-06-20

**Authors:** Pedro Magalhães Ferreira, Rui Campos, Carolina Valente, Joana Ferreira, Cláudia Freitas, Catarina Sousa, David Araújo, Hélder Novais Bastos, Adriana Magalhães, Maria Gabriela O. Fernandes

**Affiliations:** 1. Departamento de Pneumologia, Centro Hospitalar Universitário de São João, Porto, Portugal.; 2. Faculdade de Medicina, Universidade do Porto, Porto, Portugal.; 3. Instituto de Patologia Molecular e Imunologia, Universidade do Porto, Porto, Portugal.

**Keywords:** Lung cancer, Metastasis, Lymph nodes, Prognosis, Survival

## Abstract

**Objectives:**

The 9th edition of the Tumor, Node, Metastasis (TNM-9) lung cancer classification is set to replace the 8th edition (TNM-8) starting in 2025. Key updates include the splitting of the mediastinal nodal category N2 into single- and multiple-station involvement, as well as the classification of multiple extrathoracic metastatic lesions as involving a single organ system (M1c1) or multiple organ systems (M1c2). This study aimed to assess how the TNM-9 revisions affect the final staging of lung cancer patients and how these changes correlate with overall survival (OS).

**Methods:**

This retrospective cohort study included patients diagnosed with lung cancer between 2018 and 2021, who were staged according to both TNM-8 and TNM-9 criteria. The staging classifications were analyzed and compared in relation to OS.

**Results:**

Among a total of 914 patients, 42 were re-staged using TNM-9. Of the 382 patients classified as stage IVB, 55.9% were reclassified as M1c2. Despite an absolute increase in mean OS for patients re-staged from IIB to IIA and from IIIA to IIB, the observed differences were not statistically significant. Median OS differed significantly both within stage IVB and between patients with M1c2 disease and other stage IV subgroups. Multi-organ metastatic disease was an independent predictor of poorer OS, regardless of age, sex, performance status, and oncologic treatment.

**Conclusions:**

TNM-9 improves prognostic accuracy in lung cancer. Although patients with multiple extrathoracic metastases involving different organ systems are not yet independently staged from IVB, they demonstrated significantly poorer OS compared to other advanced-stage patients.

## INTRODUCTION

Despite significant efforts in both prevention and screening, lung cancer remains the leading cause of cancer-related death worldwide.^1^ One major area of advancement in lung cancer management is the optimization of staging systems, which aim to more accurately predict which patients are best suited for specific treatment modalities. 

Since 1966, the well-established Tumor, Node, Metastasis (TNM) classification has been used to stage cancer patients based on the anatomic extent of malignancy-both to guide treatment decisions and to serve as a prognostic indicator. Recent updates have been driven by large-scale, prospective research led by the International Association for the Study of Lung Cancer (IASLC).^2^ The 9th edition of the TNM staging system (TNM-9), set to become standard practice as of January 2025, is based on comprehensive analyses of international lung cancer databases compiled by the IASLC. As in previous editions, TNM-9 categorizes tumors according to primary tumor characteristics (T), the presence or absence of regional lymph node involvement (N), and the presence or absence of distant metastases (M).^3^ The proposed changes in TNM-9 include the subdivision of the former mediastinal nodal category (N2) into single-station (N2a) and multiple-station (N2b) involvement, as well as the reclassification of multiple extrathoracic metastatic lesions (M1c) into either single-organ system (M1c1) or multiple-organ system (M1c2) categories.^4^ While the N2a/N2b distinction may alter a patient’s final stage compared to the 8th edition (TNM-8), the new subcategorization of M1c patients does not yet alter the overall stage-all such cases remain classified as stage IVB.

In this study, we aimed to evaluate how the application of the novel TNM-9 staging criteria affects the final classification of lung cancer patients previously staged using TNM-8, and how these changes may translate into differences in overall survival (OS).

## METHODS

This retrospective cohort study included all patients diagnosed with lung cancer at a tertiary center between January 2018 and December 2021. Data were manually extracted from the electronic health records of patients treated at our institution. Patients with insufficient clinical information were excluded from the final analysis (see Supplementary Figure 1). Additionally, individuals diagnosed and treated at another facility or referred to our center solely for treatment were also excluded. To ensure patient confidentiality, no personally identifiable information was collected.

**Figure 1:**
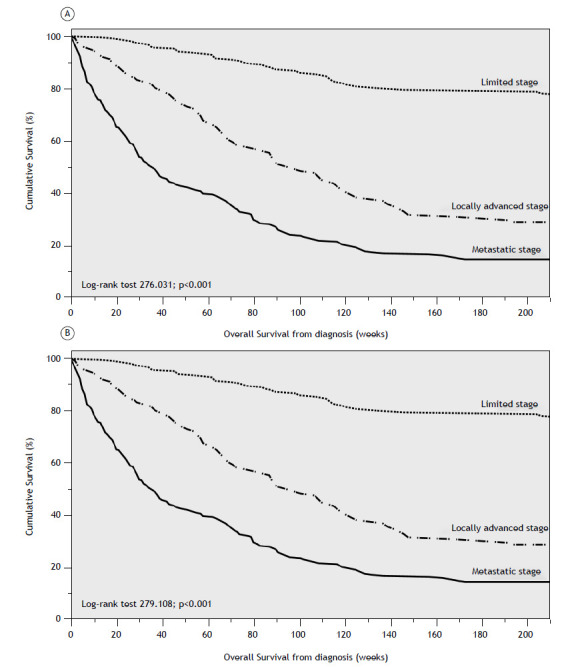
Kaplan-Meier curves showing the cumulative survival of the overall sample stratified by general staging classification (limited, locally advanced, and metastatic stages): TNM-8 (A) and TNM-9 (B).

All patients were staged either clinically or, when applicable, pathologically (after surgery), in accordance with international guidelines. Aside from the data required for complete TNM staging-primary tumor size, lymph node involvement, and presence of metastases-additional variables collected included sex, age at diagnosis, smoking status, baseline Eastern Cooperative Oncology Group Performance Status (ECOG-PS), lung cancer-related symptom status (symptomatic or asymptomatic), and patient referral setting. Information regarding first-line treatment, clinical status (alive or deceased), and overall survival (OS) was also gathered. Each patient was staged according to both TNM-8 and TNM-9 criteria. In order to stratify lymph node involvement according to the novel TNM-9 system, all histopathological results obtained from endobronchial ultrasound (EBUS) sampling were reviewed. Imaging data-including computed tomography (CT), positron emission tomography-CT (PET-CT), and brain magnetic resonance imaging (MRI)-were reassessed alongside baseline multidisciplinary tumor board reports to reclassify metastatic involvement.

Statistical analysis was performed using SPSS version 28.0 (SPSS Inc., Chicago, IL, USA). The normality of variable distribution was assessed using the Kolmogorov-Smirnov test. Categorical variables were compared using the chi-square test, and the strength of associations was measured using Cramer’s V. Survival analyses were conducted using the Kaplan-Meier method (log-rank tests), and multivariate analysis was performed using the Cox proportional hazards model.

This study was carried out in accordance with the Declaration of Helsinki and was approved by the Ethics Committee of the São João Local Healthcare Unit, in Porto, Portugal (CES ULSSJOAO: 21/2024). Due to its retrospective nature, the requirement for individual informed consent was waived by the committee.

## RESULTS

A total of 914 patients were diagnosed with lung cancer during the study period. The majority were male (71.7%), and the mean age at diagnosis was 67.72 (± 10.59) years. Most patients exhibited either active or former smoking habits (77.7%) and were symptomatic at the time of diagnosis (59.8%). Patients were primarily referred from outpatient settings, either following consultation with a general practitioner (35.6%) or a hospital-based consultation (32.2%). The median ECOG-PS at diagnosis was 0 (range: 0-4). The most common histological subtype was lung adenocarcinoma (n=570; 62.4%), followed by squamous cell carcinoma (n=167; 18.3%) and small cell lung cancer (n=98; 10.7%). Additional demographic data are presented in Table 1.

**Table 1:** Baseline characteristics of the study population.

	N = 914
Age (years)	67.8 ± 10.6
Sex (male)	655 (71.7)
Smoking status	
Non-smoker	204 (22.3)
Former smoker	312 (34.1)
Active smoker	398 (43.5)
Referral setting	
Primary healthcare	325 (35.6)
Outpatient clinic	294 (32.2)
Hospital ward	223 (24.4)
Emergency department	72 (7.9)
ECOG Performance Status	
0-1	730 (79.9)
2-4	184 (20.1)
Histological subtype	
Adenocarcinoma	570 (62.4)
Squamous cell carcinoma	167 (18.3)
Small cell lung cancer	98 (10.7)
Carcinoid tumor	33 (3.6)
NSCLC-NOS	29 (3.2)
Large cell carcinoma	17 (1.9)
Oncologic treatment	
Chemotherapy	229 (25.1)
Surgery (VATS)*	202 (22.1)
Chemoradiation therapy	189 (20.7)
Stereotactic body radiation therapy	85 (9.3)
Target therapy	68 (7.4)
Immune checkpoint inhibitor therapy	53 (5.8)
Chemoimmunotherapy	26 (2.8)
Hormone therapy	1 (0.1)
Watchful waiting/Best supportive care	61 (6.7)
Mortality	
Overall mortality	494 (54)
12-month mortality	342 (37.4)

Legend: ECOG, Eastern Cooperative Oncology Group; NSCLC-NOS, Non-small cell lung cancer not otherwise specified; VATS, Video-assisted thoracoscopic surgery. Continuous variables presented as mean ± standard deviation; qualitative variables presented as absolute number (percentage).

The complete TNM staging classifications for the 8th (TNM-8) and 9th (TNM-9) editions are presented in Supplementary Table 1. Based on TNM-8, 270 patients (29.5%) were classified as having limited-stage disease, whereas under TNM-9 criteria, this number increased slightly to 279 (30.5%). This shift is attributable to the downstaging of 9 patients who had previously been classified as having locally advanced disease under TNM-8 (261 patients vs. 252 under TNM-9). As expected, the number of advanced-stage patients remained unchanged between editions (n=383; 41.9%). Overall, this resulted in a significant, strong, and positive correlation between the two staging systems (p=0.003; V=0.983). An in-depth analysis revealed that 34 patients were downstaged and 8 were upstaged when transitioning from TNM-8 to TNM-9. Table 2 details these changes in final clinical staging. Downstaging occurred at three levels: 6 patients from IIB to IIA, 9 from IIIA to IIB, and 19 from IIIB to IIIA. Upstaging, on the other hand, involved 8 patients reclassified from IIIA to IIIB. The TNM-8 N2 subgroup (n=237) was further subdivided in TNM-9 into N2a (n=115; 48.5%) and N2b (n=122; 51.5%). Among the 382 advanced-stage patients, 290 (75.9%) were classified as stage IVB (M1c) due to multiple metastases. Under the updated TNM-9 criteria, although no change occurred in overall staging due to the current classification structure, 128 patients (44.1%) were categorized as M1c1 (single-organ system involvement), and 162 (55.9%) as M1c2 (multi-organ system involvement). The distribution of primary metastatic sites by TNM-9 M subclassification is provided in Supplementary Table 2.

**Table 2:** Re-staged lung cancer patients after TNM-9 staging criteria.

	DOWNSTAGED		UPSTAGED		
	IIB to IIA (n=6)	IIIA to IIB (n=9)	IIIB to IIIA (n=19)	IIIA to IIIB (n=8)	IVB* (n=290)
Primary tumor size					
T1a	1 (16.7)	1 (11.1)	0 (0)	0 (0)	14 (4.8)
T1b	3 (50.0)	6 (66.7)	0 (0)	0 (0)	22 (7.6)
T1c	1 (16.7)	2 (22.2)	0 (0)	0 (0)	28 (9.7)
T2a	1 (16.7)	0 (0)	0 (0)	6 (75.0)	41 (14.1)
T2b	0 (0)	0 (0)	0 (0)	2 (25.0)	19 (6.6)
T3	0 (0)	0 (0)	19 (100)	0 (0)	58 (20.0)
T4	0 (0)	0 (0)	0 (0)	0 (0)	108 (37.2)
Lymph node staging					
N1	6 (100)	0 (0)	0 (0)	0 (0)	58 (20.0)
N2a	0 (0)	9 (100)	19 (100)	0 (0)	40 (13.8)
N2b	0 (0)	0 (0)	0 (0)	8 (100)	46 (15.9)
N3	0 (0)	0 (0)	0 (0)	0 (0)	146 (50.3)
Metastatic disease					
IVB (M1c1)	0 (0)	0 (0)	0 (0)	0 (0)	128 (44.1)
IVB (M1c2)	0 (0)	0 (0)	0 (0)	0 (0)	162 (55.9)

Legend: TNM-9, Tumor, Node, Metastasis classification for lung cancer (9th edition). Qualitative variables presented as absolute number (percentage). *Not an actual re-staging, but an updated differentiation between multiple metastatic lesions in a single organ system (M1c1) versus in more than one organ system (M1c2).

The median OS (mOS) at diagnosis was 96 weeks (95% CI: 83.99-108.01) and differed significantly by overall stage (limited, locally advanced, and advanced) under both TNM-8 and TNM-9 criteria (Figure 1). 

**Figure 2:**
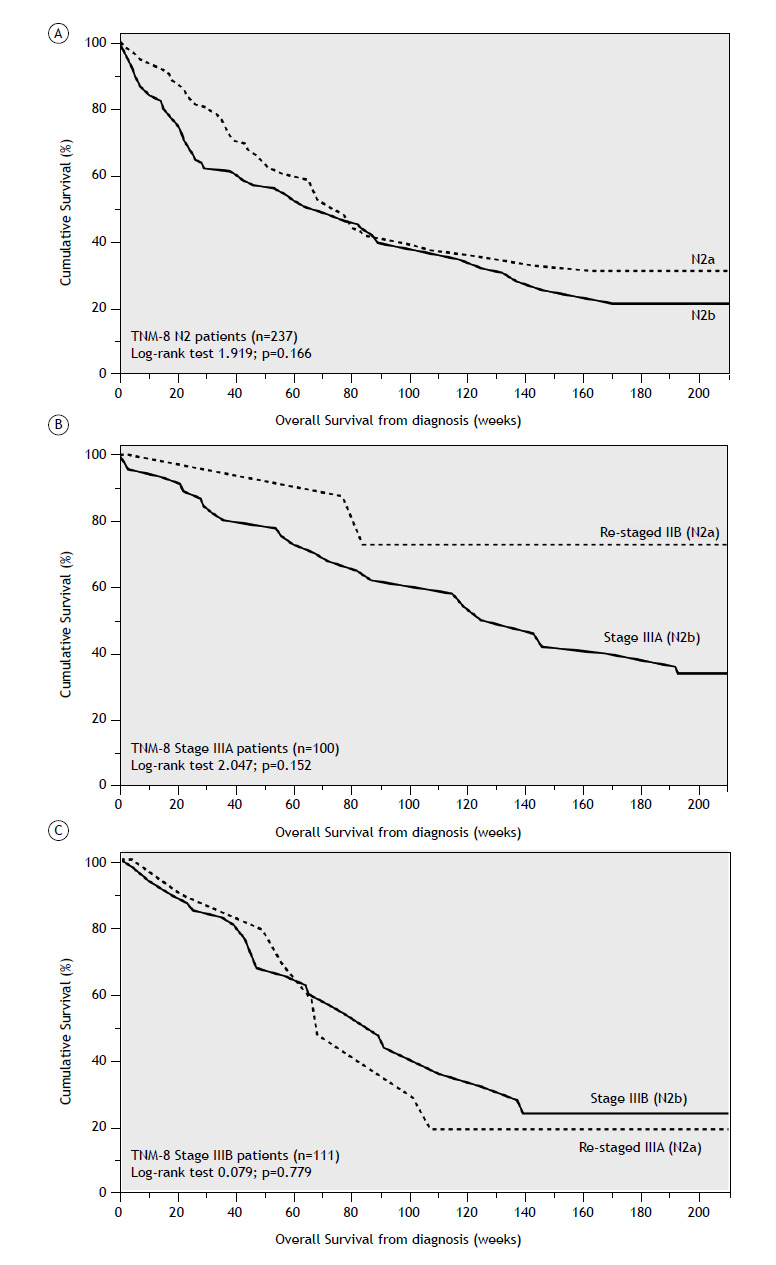
Kaplan-Meier curves showing the cumulative survival based on lymph node involvement after TNM-9 reclassification: overall N2 involvement (A); TNM-8 stage IIIA patients (B); TNM-8 stage IIIB patients (C).

Stratification by N status revealed statistically significant differences in mOS under both staging systems. While patients with N1 and N3 disease showed similar survival patterns, TNM-8 N2 patients had a mOS of 72 weeks (95% CI: 61.62-82.38) after diagnosis. Under TNM-9, this group was subdivided into N2a [mOS 74 weeks (95% CI: 63.32-84.68)] and N2b [mOS 71 weeks (95% CI: 47.97-94.03)]. Among patients staged between IIA and IIIC, survival differed significantly according to stage, regardless of the TNM edition applied. Although the overall mOS across both editions was identical (110 weeks, 95% CI: 93.05-126.95), a notable difference was observed in stage IIIA: TNM-8 stage IIIA patients had a mOS of 143 weeks (95% CI: 114.28-171.72), compared to 119 weeks (95% CI: 95.71-142.29) for TNM-9 stage IIIA patients. No survival differences were observed between TNM-8 and TNM-9 in stages IIIB and IIIC. In patients downstaged under TNM-9, mOS was not reached due to high survival rates at the time of the analysis. However, mean survival times were higher for patients downstaged from IIB to IIA and from IIIA to IIB, though not for those downstaged from IIIB to IIIA (Figure 2). Despite these absolute increases in survival, the differences were not statistically significant.

**Figure 3:**
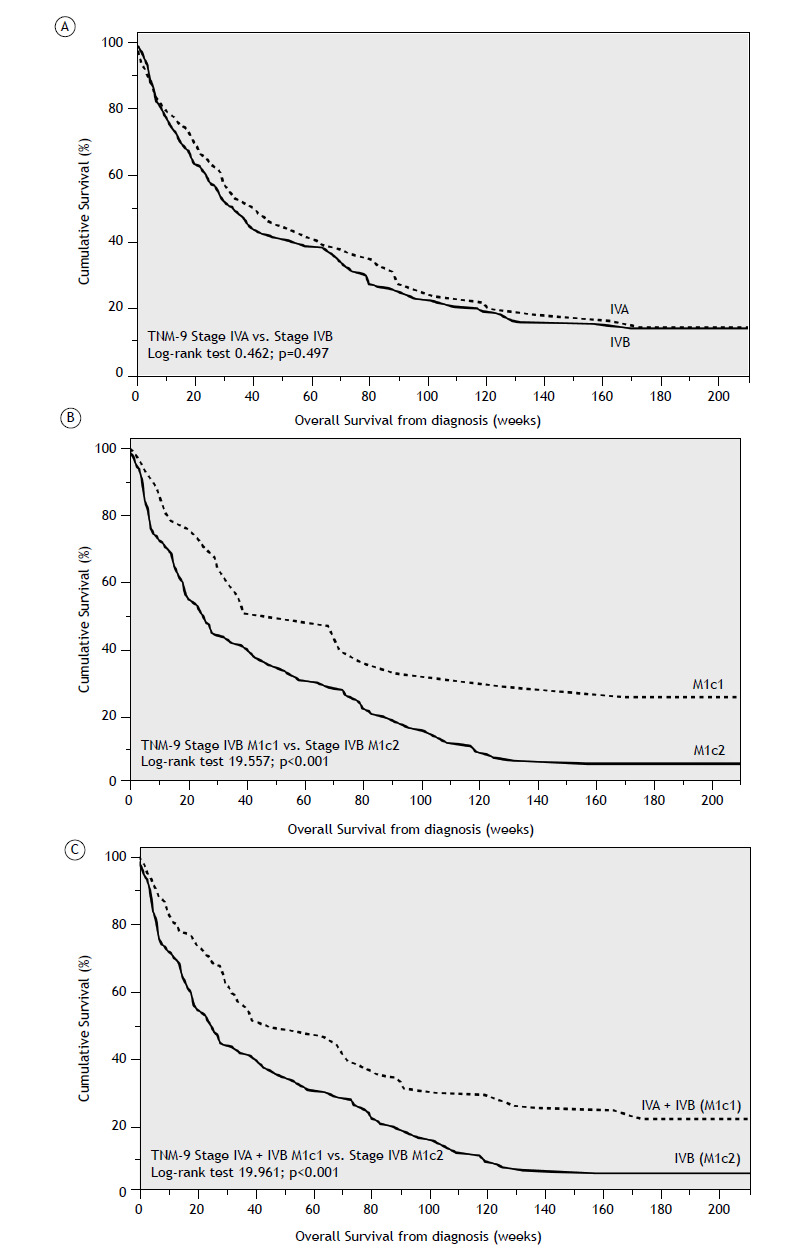
Kaplan-Meier curves showing the cumulative survival of patients with metastatic disease: stage IVA versus IVB (A); stage IVB patients stratified by M1c1 versus. M1c2 (B); M1c2 patients (multiple metastases in >1 organ system) versus all other stage IV patients (C).

OS did not differ significantly between stages IVA and IVB under either classification system (p=0.497). However, specific metastatic sites-particularly the liver and adrenal glands-were associated with significantly poorer prognoses. Patients with liver metastases had a mOS of 4 months (95% CI: 0.71-7.29), compared to 9 months (95% CI: 6.81-11.19) in those without liver involvement (p<0.001). Similarly, patients with adrenal metastases had a mOS of 4 months (95% CI: 1.56-6.44) versus 8 months (95% CI: 5.95-10.01) in those without adrenal involvement (p=0.011). When stratified by the M1c classification (Figure 3), M1c2 patients had significantly shorter survival than those with M1c1 disease [25 weeks (95% CI: 18.82-31.18) vs. 68 weeks (95% CI: 44.18-91.82); (p<0.001)], as well as when compared to all other M1 patients combined (i.e., stage IVA and non-M1c2 stage IVB) [mOS of 42 weeks (95% CI: 24.24-59.76); p<0.001]. No other statistically significant differences in OS were observed across metastatic stages. In a multivariate analysis restricted to stage IVB patients (Table 3), including variables such as M1c subclass (M1c1 vs. M1c2), sex, age at diagnosis, smoking status, ECOG-PS, specific metastatic sites (brain, bone, pleura), and treatment modality (systemic therapy vs. best supportive care), the M1c2 status remained an independent predictor of poorer OS [HR 1.43 (95% CI: 1.03-1.97); p=0.031], as did worse baseline ECOG-PS [HR 1.81 (95% CI: 1.26-2.60); p=0.001].

**Table 3:** Adjusted survival analysis of stage IVB lung cancer patients according to the updated TNM-9 classification - univariate and multivariate logistic regression analyses.

	Univariate Analysis		Multivariate Analysis	
	Adjusted HR (95% CI)	p-value	Adjusted HR (95% CI)	p-value
Sex (male)	1.18 (0.89-1.58)	0.246	1.29 (0.89-1.85)	0.172
Age at the time of diagnosis	1.02 (1.01-1.04)	<0.001	1.01 (0.99-1.03)	0.071
Smoking (active/former smoker)	1.63 (1.18-2.25)	0.003	1.31 (0.85-2.01)	0.225
ECOG Performance Status (2-4)	2.63 (1.99-3.48)	<0.001	1.81 (1.26-2.60)	0.001
Specific metastatic sites				
Brain metastasis	1.03 (0.78-1.37)	0.818	1.20 (0.85-1.69)	0.291
Pleural metastasis	1.34 (0.96-1.86)	0.085	1.28 (0.88-1.87)	0.202
Bone metastasis	0.87 (0.67-1.13)	0.298	0.99 (0.72-1.38)	0.983
Treatment (Systemic vs. BSC)	0.17 (0.12-0.26)	<0.001	0.35 (0.22-0.58)	<0.001
Multiple metastasis in >1 organ system (M1c2)	1.83 (1.39-2.40)	<0.001	1.43 (1.03-1.97)	0.031

Legend: BSC, Best supportive care; CI, Confidence interval; ECOG, Eastern Cooperative Oncology Group; HR, Hazard ratio; TNM-9, Tumor, Node, Metastasis classification for lung cancer (9th edition).

## DISCUSSION

The application of the novel TNM-9 lymph node staging criteria resulted in the downstaging of 34 patients-primarily from stage IIIB to IIIA-and the upstaging of 8 patients from IIIA to IIIB. Although downstaged patients showed a trend toward improved OS when compared to those whose stage remained unchanged regardless of the TNM edition, no statistically significant differences in OS were observed following N2 re-stratification. The new TNM-9 subclassification of M1c patients revealed significantly different OS outcomes between single-organ and multi-organ metastatic disease. However, this distinction itself is not yet reflected in final stage grouping, as both M1c1 and M1c2 remain classified as stage IVB.

The rationale behind introducing a new stratification model for nodal disease in TNM-9 stems from previous reports suggesting poorer prognoses associated with either the number of involved lymph nodes^5^ or the number of nodal stations affected.^6^ ^,^ ^7^ It is important to note, however, that in the 9th edition database, the prognostic distinction between N2a and N2b was primarily established through imaging techniques (CT, PET-CT) used to determine clinical stage,^8^ rather than through invasive procedures such as EBUS. This contrasts with the present study, in which each patients’ histopathological record was reviewed and re-staged based on transbronchial needle aspiration (EBUS-TBNA). Lymph node sampling is arguably the most significantly impacted component of staging with the implementation of TNM-9.^9^ EBUS remains the most widely adopted method for lymph node staging in lung cancer, and-together with transesophageal endoscopic ultrasound (EUS/EUS-b)-is currently recommended over mediastinoscopy for pathological mediastinal staging.^10^ ^,^ ^11^ Traditionally, EBUS-based staging begins with any N3 station (if present), subsequently proceeding to more proximal lymph node stations (N2, N1). PET-CT and ultrasound features, such as lymph node size, uptake, and vascularity, can help determine which N2 lymph nodes are most likely malignant and should be prioritized for sampling.^12^ ^-^ ^14^ With the introduction of the TNM-9 classification, however, due to the staging implications related to N2a/N2b differentiation, it is now mandatory to sample all ipsilateral mediastinal and subcarinal stations. Moreover, other lymph node stations-including the superior paratracheal (2L and 2R), paraoesophageal,^8^ and pulmonary ligament nodes^9^-are expected to become part of routine systematic staging. This expanded approach not only increases overall procedure duration but also raises the cost of lymph node staging. While the principle of sampling N3 stations first and progressing to N2 and N1 remains valid, sampling multiple N2 stations may require changing needles to avoid cross-contamination. In our study, we found no significant difference in OS when stratifying N2 patients with pathological confirmation of single- versus multi-station involvement. Prospective studies will be essential to determine whether the additional effort required for the new nodal stratification ultimately results in meaningful improvements in treatment decision-making and patient outcomes.

In contrast to lymph node staging, the changes introduced by TNM-9 in the assessment of metastatic disease did not alter the overall classification of stage IV patients.^15^ Although some authors have suggested that subdividing M1c into M1c1 (multiple extrathoracic metastases within a single organ system) and M1c2 (multiple extrathoracic metastases across multiple organ systems) is relevant for refining prognoses,^4^ ^,^ ^8^ the lack of a distinct final stage classification results in the impact of multi-organ dissemination being diluted within the broader IVB category. Our study indicates a significant difference in clinical outcomes for patients with M1c2 disease, both within the IVB subgroup and compared to all other stage IV patients. This finding is supported by several studies on lung cancer metastasis, which highlight the impact of a higher metastatic burden on overall prognosis.^16^ ^,^ ^17^ Notably, the survival difference between M1c2 and other stage IV patients was greater than that observed between M1a and the rest of the stage IV population, underscoring the importance of identifying this subset for both accurate prognostication and potential treatment stratification. Overall, these findings are consistent with the evolving understanding of oligometastatic disease, a concept first introduced in 1995.^18^ Although no universally accepted definition exists, oligometastatic disease generally refers to a limited number of metastatic lesions in a limited number of organs that may be amenable to curative local therapy.^19^ While some debate persists over the false notion that metastatic disease follows bimodal distribution (oligometastatic vs. multi-metastatic) instead of a disease continuum,^20^ defining oligometastasis holds clinical relevance in the sense that it may guide the use of different, curative-intent treatment modalities in this specific context.^21^ ^,^ ^22^ Therefore, clearly delineating the prognostic distinction between patients potentially classified as oligometastatic (M1a, M1b, and M1c1) and those unlikely to benefit from more radical treatment approaches (M1c2) becomes increasingly important in the era of patient-tailored medicine.^23^ Although the novel TNM-9 classification represents a step forward from TNM-8, it falls short in refining the staging of advanced-stage lung cancer by failing to independently classify M1c2 patients and by not fully addressing the prognostic differences observed in this subgroup.

This study has some limitations. First, it was a retrospective, single-center study, inherently dependent on the quality of available clinical data. Reports of N2 lymph node involvement relied on procedures performed before the implementation of the novel staging recommendations. Reclassification into N2a/N2b was based on histopathological confirmation of multi-nodal disease via EBUS-TBNA; however, these procedures may not have adhered to the revised systematic assessment of all relevant lymph node stations. Additionally, since this is not yet standard practice, TBNA needles were likely not changed between N2 stations, introducing the potential for cross-contamination. Although our dataset is smaller than those used by the IASLC, it represents a substantial real-word sample. This difference may explain the lack of statistical significance observed following N2a/N2b reclassification, as only 42 patients were affected by the updated criteria. Nevertheless, with regard to advanced-stage disease in particular, we believe our data are robust enough to support a discussion about the lack of practical distinction between other stage IVB patients and those reclassified as M1c2-who may be more appropriately staged as IVC. Future prospective studies should focus on assessing the clinical impact of the novel N2 classification and establishing prognostic thresholds for M1c2 patients to determine whether they should be staged separately.

In conclusion, the updated TNM-9 staging guidelines introduce significant changes to both the lymph node involvement and metastatic disease components of the previous edition, enhancing prognostic accuracy for patients with lung cancer. Although M1c2 patients-those with multiple extrathoracic metastases involving multiple organ systems-are not classified separately within stage IV disease, they exhibited significantly poorer OS rates compared to other advanced-stage patients. This finding highlights the need for future discussions on prognostic stratification and treatment strategies within the context of disseminated disease.
